# Peatlands management for sustainable use on the integration of maize and cattle in a circular agriculture system in West Kalimantan, Indonesia

**DOI:** 10.1016/j.heliyon.2024.e31259

**Published:** 2024-05-16

**Authors:** Dwi P. Widiastuti, Muhammad Hatta, Hozin Aziz, Dadan Permana, Putri Tria Santari, Eni Siti Rohaeni, Salfina Nurdin Ahmad, Bachtar Bakrie, Siti Sehat Tan, Susana I.W. Rakhmani

**Affiliations:** a Research Center for Food Crop, Research Organization for Agriculture and Food, National Research and Innovation Agency of Indonesia (BRIN), Soekarno Science and Technology Center, Jl. Raya Jakarta-Bogor KM 46, Cibinong, West Java, 16911, Indonesia; b Research Center for Appropriate Technology, Research Organization for Agriculture and Food, National Research and Innovation Agency of Indonesia (BRIN), Subang Science Center, Jl. K.S. Tubun 5, Subang, West Java, 41213, Indonesia; c Research Center for Cooperatives, Corporations, and People's Economy, Research Organization for Governance, Economy, and Community Welfare, National Research and Innovation Agency of Indonesia (BRIN), Gedung Sasana Widya Sarwono BRIN, Lantai 03 Jl. Gatot Subroto 10, Jakarta, 12710, Indonesia; d Research Center for Animal Husbandry, Research Organization for Agriculture and Food, National Research and Innovation Agency of Indonesia (BRIN), Soekarno Science and Technology Center, Jl. Raya Jakarta-Bogor KM 46, Cibinong, West Java, 16911, Indonesia; e Research Center for Social Welfare, Villages and Connectivity, Research Organization for Governance, Economy, and Community Welfare, National Research and Innovation Agency of Indonesia (BRIN), Gedung Sasana Widya Sarwono BRIN, Lantai 03 Jl. Gatot Subroto 10, Jakarta, 12710, Indonesia

**Keywords:** Peatland management, Circular agriculture, Rural, Maize-cattle integration, Institutional engineering, Increased income, Environmentally friendly, Sustainable

## Abstract

Management of peatlands with an integrated system of maize and cattle is an alternative to circular agriculture in peatland areas that can increase economic growth in rural areas which is environmentally friendly and sustainable (green economy). This study aims to obtain a circular agriculture model with zero waste management based on the integration of maize and cattle which is environmentally friendly and sustainable in peatland areas, in addition to increasing the added value and farmers' income. This circular agriculture model utilizes site-specific technological innovations and local resources that can restore sustainability in the peatland. The analysis methods used were before-and-after study analysis, baseline surveys, field trials using an experimental design and analysis of variance, financial analysis, and institutional engineering. To achieve this goal, two sub-models were implemented, namely a rural agro-industrial approach based on local agricultural resources by managing appropriate site-specific technological innovations (on-farm) and a local human resource approach through rural institutional engineering, that is farmers’ institutional initiation, development, empowerment, and institutional strengthening of agribusiness (off-farm). The results showed that the circular rural agricultural management model on integrating maize and cattle as a benchmark could increase farmers' income in the peatland areas by more than 208 % from IDR 4,760,000 to IDR 14,600,000 per month. The management of peatlands through circular agriculture can improve quality products and add value to the utilization of waste such as animal feed products (silage), organic fertilizers, and biourine. This rural circular agriculture model is carried out by social engineering, initiation, and strengthening of rural agribusiness institutions that are environmentally friendly so that they can be sustainable.

## 1 Introduction

Indonesia will face food security problems due to reduced rice fields area and the threat of global climate change through climate anomalies and environmental challenges. The area of paddy fields in Indonesia has decreased from 7.7 million hectares in 2013 to 7.4 million hectares in 2018 [1]. The area of paddy fields that tend to decline needs to be observed carefully, among others, by increasing the productivity of alternative lands that have the potential for agricultural areas, such as peatlands. The area of peatlands in Indonesia in 2020 is 13.43 million hectares, potentially for food crop development [2].

Peatlands are land with relatively low productivity, so there are still many obstacles and problems for food crop cultivation. These constraints include the presence of pyrite compounds (FeS_2_), high iron and aluminum solubility, low pH, nutrient deficiency, high organic acid concentrations, small nutrient concentrations of K, S, Zn, and Cu that are not available for plants [[3], [4], [5], [6], [7]].

However, with prudent actions and specific technologies that are environment-friendly, peatlands can be utilized for sustainable agricultural areas, among others, by establishing land use zoning, improving land and crop management systems, and as importantly efforts to increase added value, strengthen institutions and policy support [8,9]. The Food and Agriculture Organization (FAO) recommends that peatland must be managed in a responsible, environment-friendly, and sustainable approach, and to reduce the negative impact of greenhouse gas emissions in the peatland areas [10].

Meanwhile, the existing condition of low-productivity peatlands will have implications for food availability and the income of family farms living in the peatland areas. Food sources of family farms are from self-produced or purchased food. Sufficient food availability for family farms is influenced by optimal land productivity, while food purchase requires sufficient income. This marginal peatland condition has the potential to trigger low welfare of the family farms. Therefore, technological innovation is required in order to increase peatland productivity and the added value of farming, specifically with an integrated circular agriculture system that can increase family farms income and is environment-friendly and sustainable in the peatland areas. The Indonesian government has changed the traditional linear economy concept to the circular economy concept [11]. This circular economy concept can be applied in rural areas, among others, with a circular agriculture system which utilizes local rural resources with zero waste or waste minimization and is sustainable.

The integration system between maize and cattle can be used as a model for circular agriculture systems in rural peatland areas. The circular agriculture model is an integration system that contributes to each other and mutually utilizes its waste products (zero waste). Maize farming contributes its yield and waste to animal feed ingredients, while cattle farming contributes to maize farming in terms of livestock labor and waste products for fertilizer. This can answer problems such as high cost of agricultural production, inefficient farming, unavailability of animal feed in the dry season, and fertilizer unavailability in the growing season. Meanwhile, family farms in the peatland areas are challenged with two interests, namely maximizing their income, and maintaining environmental quality, including greenhouse gas emissions in the peatlands. In order to tackle these conditions, a circular agriculture system based on the integration of maize and cattle is needed to develop in a profitable, environment-friendly, and sustainable way.

One potential integration system as a circular economy concept is the integration of maize and cattle that have developed with various variations and configurations, in accordance with the level of technology uptake and institutional enrichment in rural areas. However, the rural circular agriculture system in Indonesia has not been applied optimally because it has weaknesses including not optimally utilizing biotechnology, mechanization, integration, and farmer institutional management in providing qualified products and derivative products in a circularly sustainable way.

West Kalimantan Province is relevant for the development of circular agriculture because it has peatland resources and local resources with local wisdom that have the potential to develop an integrated circular agriculture system. The area of peatlands in West Kalimantan is around 1.5 million hectares [2], while the planting area of maize is 53,473 ha and the cattle population is 159,100 [12]. The extent of corn plants has the potential for waste from stalks, straw, and corn cobs as animal feed and the high population of cattle as a source of organic fertilizer. This is one of the solutions to solve the problems of regional and national food security which is environment-friendly by producing maize, beef, and their derivative products. This needs to be done with the support of technological and institutional innovation so that an agricultural system with a circular and sustainable economy concept can be achieved. Until now, research on crops and livestock has only been limited to biophysical research on land and livestock, it has not been carried out comprehensively and it has not involved institutional management in rural areas. Therefore, the results of peatland management research for the development of circular agriculture based on the integration of maize and cattle can respond to the problems in rural areas. The resulting model can be used as a recommended circular agriculture system with zero waste, increasing income, and being environment-friendly and sustainable.

Research on peatland management for the development of circular agriculture based on the integration of maize and cattle aims to obtain a circular agriculture system model with zero waste that can increase added value and farmer's income that is environment-friendly and sustainable in the peatland areas.

## 2 Methodology

### 2.1 Location and timeline of research

Research on peatland management for the development of rural circular agricultural systems based on the integration of maize and cattle was carried out in the maize center area in the peatland area of Rasau Jaya Dua Village, Rasau Jaya District, Kubu Raya Regency, West Kalimantan Province, Indonesia. The location was determined based on the results of land use zoning that was carried out in the previous year [13]. This research project activity was conducted for four years from 2016 to 2020, starting from preliminary surveys, participatory rural appraisal, on-farm and off-farm research, land biophysical analysis, animal feed, manure, farmer institutional analysis, and farming analysis. This research involves several multidisciplinary experts and cooperative farmers that makes coordination for the final report on project activities completed in early 2022. Scientific manuscript was written and submitted to the journal in 2023.

### 2.2 Materials and methods of analysis

Materials used are for baseline survey activities, initiation of farmer institutions, and the research of maize and cattle integration, such as quality maize seeds, fertilizers, and animal feed. Whereas tools used are for maize and cattle waste processing, machines for animal feed and biofertilizers, as well as other supporting tools.

This research is characterized by increased productivity of maize, increased numbers of cattle, zero waste, and maize and cattle waste processing into complementary (circular), environment-friendly, and sustainable products. The application of the circular agricultural development model is carried out by applying the principles of a sustainable rural circular economy as an integrated green economic system.

The research involved farmer groups association and a farmer group in Rasau Jaya Dua Village by implementing two sub-models, namely: 1) rural circular agriculture sub-model based on crop and livestock integration by applying site-specific technological innovation, local wisdom, and environment-friendly and 2) sub-model on institutional engineering of rural agribusiness. The research on rural circular agriculture sub-model was carried out on an integrated farming system of maize and cattle on the peatland, which is an on-farm activity. The sub-model of rural agribusiness institutional engineering is an off-farm activity, including the initiation of farmer institutions, coaching, empowerment, service, and strengthening of agribusiness institutions at the rural level in the peatland areas.

#### 2.2.1 Initiation and strengthening of farmer institutions

Initiation and strengthening of farmer institutions use descriptive analysis of the baseline survey results, namely collecting initial information about the condition of various variables in a systematic study. This analytical method is used as an indicator for initiating and strengthening institutions at the farmer or farmer groups level. In addition, this method is also used to obtain existing data on the characteristics of land resources and human resources, socio-economic conditions in rural areas, local specific resources, and farm household income. The survey results are used as a benchmark for initiating and strengthening farmer institutions in implementing the rural circular agriculture model in a sustainable maize and cattle integration system in the peatland areas. Selected farmers in the survey were representatives of the farming community with the criteria as follows: administrators and/or members of farmer groups, raising cattle, cultivating maize, cooperative farmers, non-cooperative farmers for comparison.

#### 2.2.2 Increased productivity of maize

The research on increasing maize productivity was conducted on a maize field trial in the peatlands. The analytical method was analysis of variance using Randomized Complete Block Design with two factors, namely biofertilizers (biostimulant seaweed extract (3 % extract concentration) and mycorrhiza) as the first factor and the second factor was maize varieties (Sukmaraga and Bisi 22). The experimental plot of 15 m^2^ (3 × 5 m) was planted with maize seeds with a spacing of 75 × 20 cm. The experiment was repeated five times and continued with a significant difference test [14]. Seed treatment used mycorrhiza, in which each 5 kg of maize seed was mixed with 500 g of mycorrhiza. Seaweed extract was prepared using the Briceño-Domínguez, Hernández Carmona, Moyo, Stirk, & van Staden method [15,16].

In addition, the application of 300 kg ha^−1^ NPK Phonska, 100 kg ha^−1^ Urea, and 50 kg ha^−1^ SP-36 as essential fertilizers. The experimental parameters were plant height, cob weight, seed weight per ear, and dry harvested weight converted into tons per hectare.

#### 2.2.3 Maize waste processing as animal feed

**Fig. 1:**
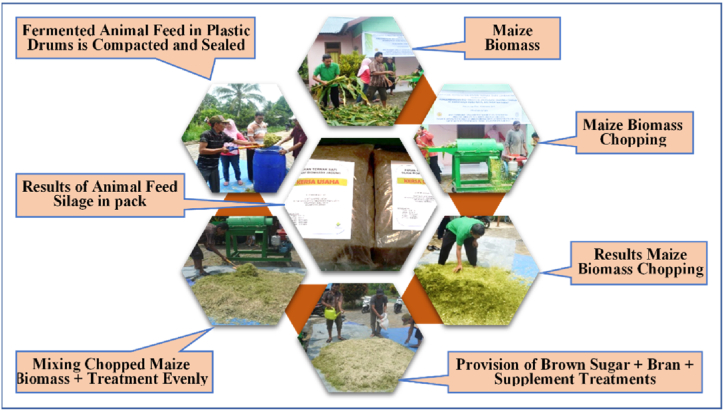
Fermentation process of animal feed from maize waste.

Processing of maize waste as animal feed was carried out through a fermentation process. Before fermentation, the maize stover was sun dried for 3 days until the moisture content was about 60–70 %, then chopped with a chopper machine into small-shredded fibers (Fig. 1). This fermentation method was carried out by utilizing *Trichoderma virideae* and adding animal feed supplements, brown sugar, and fine bran. The analytical method in this study used analysis of variance with a complete randomized design (CRD) and consisted of three treatments, i.e.: A) 4 kg brown sugar + 6 kg rice bran + 100 kg maize stover + 15 ml animal feed supplement, B) 3 kg brown sugar + 5 kg rice bran + 100 kg maize stover + 15 ml animal feed supplement, and C) 2 kg brown sugar + 4 kg rice bran + 100 kg maize stover + 15 ml animal feed supplement.

The fermentation method used in this study was as follows: 1) The maize stover was chopped with a chopper to make it easily broken down by bacteria so that the ensilage process run smoothly. 2) The chopped maize stover was added with some treatment, then compacted and stored in a plastic drum and tightly closed to prevent some air cavity. 3) After six weeks, the drum was opened, then the silage was dried and packaged in ready-to-use plastic bags for animal feed or to be stored (Fig. 1). The parameters observed were smell, taste, color, texture, and the presence of mold in the animal feed.

#### 2.2.4 Processing cattle waste into organic fertilizer

**Fig. 2:**
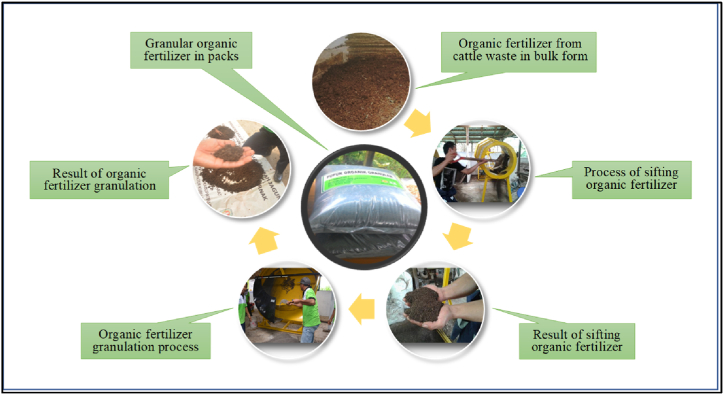
Production process of granular organic fertilizer.

Cattle waste was processed into solid and liquid organic fertilizer (biourine). Solid waste processing used the composting method by utilizing a decomposer which accelerated the decomposition process to produce solid organic fertilizer compost with high nutrient content. As for the liquid waste (cow urine) into biourine products using the fermentation method. The process of making organic fertilizer was carried out by collecting livestock waste from stables designed to facilitate the separation of solid and liquid waste (cow urine). Solid waste that was still fresh was collected in a holding tank, then mixed homogenously with a decomposer. It was stirred every 5-days and closed tightly until a mature bulk organic fertilizer was obtained by dark color, loose structure, and odorless characters. Bulk organic fertilizer was further processed into organic fertilizer in granular form (Fig. 2).

**Fig. 3:**
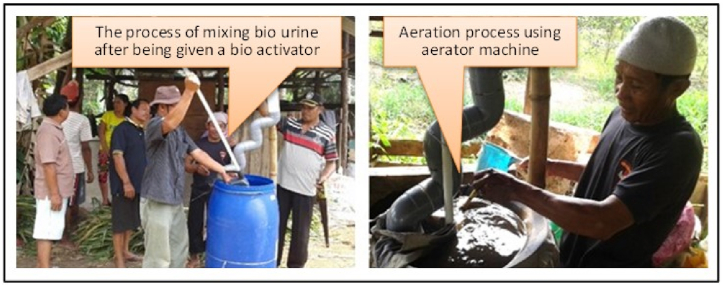
Biourine manufacturing process in Rasau Jaya Dua Village.

While the biourine was processed by adding and homogenously stirring bio-activator to 200 L cow urine in a plastic drum and then it was tightly closed. It was stirred for 5 min every 5 days and tightly closed again. The fermentation process took about 14 days. After 14 days, the aeration process was carried out by pumping urine using an aerator machine for 3 days through a channel made like a ladder from a paralon pipe (Fig. 3).

The biourine trial analysis method used an analysis of variance with a complete randomized design. The treatments were 1) cow urine + bio-activator, 2) cow urine + bio-activator + liquid herbs made from ginger, turmeric, curcuma, aromatic ginger, lemongrass, and garlic (5 % amount of all ingredients) + enzymes from the rumen (1 L enzyme per 50 L urine), 3) cow urine + bio-activator + liquid herbs made from ginger, turmeric, curcuma, aromatic ginger, lemongrass, and garlic (5 % amount of all ingredients) + enzymes from the rumen (1 L enzyme per 50 L urine) + rice washing water (100 mL rice washing water per L urine) + potassium permanganate (KMnO_4_) and titanium dioxide (TiO_2_) (2 g each per L water). The parameters observed were the smell of cow urine and nutrient content.

#### 2.2.5 Farming analysis of maize-cattle integration on the peatlands

This integrated farming analysis method was obtained based on the calculation of the total cost and revenue margin analysis from maize cultivation and cattle farming as well as the value-added from the processing of maize waste and cattle waste with the following formula: [17].

Notes:Π=TR−TVC−TFC

$$\Pi =\text{Pq}.Q-\sum_{i=1}^{n}Pi.Xi-\sum_{j=1}^{m}Kj$$

Π = Profit.

TR = Total Revenue.

TVC = Total Variable Cost.

TFC = Total Fix Cost.

Pq = Product price.

Q = Total Product.

Pi = Input price to-i.

Xi = Variable Input to-i.

Kj = Input fix to-j

i = Variable input types to-i (n in total)

j = Fix input types to-j (m in total)

#### 2.2.6 Analysis of technological feasibility

Economic feasibility was analyzed by marginal B/C ratio, which was the ratio between the total reduction in income (Total Losses) and the total increase in income (Total Gains). In simple terms, it can be formulated as follows:

$$\text{Marginal}\;B/C\;\text{ratio}=\frac{\text{Total}\;\text{gains}}{\text{Total}\;\text{losses}}$$

notes:

Total gains = increase in income.

Total losses = increase in costs.

Furthermore, an analysis is needed to study the relationship between production costs, sales volume, and revenue to determine the level of profit and farming feasibility. Break-even yield and price break-even analysis are tools that can be used to study the relationship between costs, revenues, and production volume [18]. The break-even yield and break-even price can be calculated using the following formula [19].

Break even yield = Total cost/Output price

Break even price = Total cost/Expected yield

Mathematically the above formulation can be obtained based on the following formula concept:

Profit = TR – TC

Profit = TR – (VC + FC)

Profit BEP condition = 0

∏ = TR – (VC + FC) = 0

TR = VC + FC

Pj.Q = VC + FC

$$Pj=\frac{\text{VC}+\text{FC}}{Q}$$

$$Q=\frac{\text{VC}+\text{FC}}{\text{Pj}}$$

## 3 Results and discussion

### 3.1 Types of land use

Land resource data provide spatial information about the distribution, extent, potential, suitability, and physical constraints of land use for agriculture, as well as alternative site-specific land management technologies, and can support the formation of integrated agricultural areas with circular agricuture systems. One of the efforts to obtain data and information on agricultural land resources is by conducting agro-ecological zoning (ZAE). Agro-ecological zoning can be classified in certain agro-ecosystem zones based on the similarity of natural factors (climate, terrain, and soil), farming activities, and socio-economic conditions in those areas. Farmers in an agro-ecosystem zone have similarities in challenges and technological needs.

**Fig. 4:**
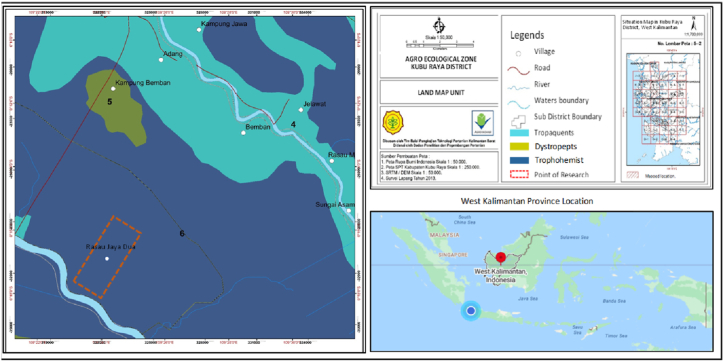
The research location in peatlands in Rasau Jaya Dua Village, Rasau Jaya Sub District, Kubu Raya District, West Kalimantan Province, Indonesia.

**Fig. 5:**
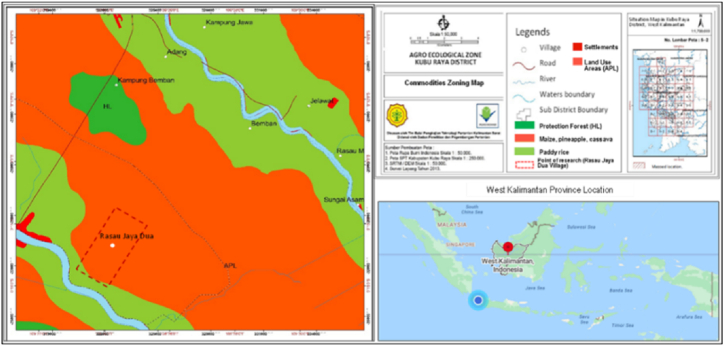
Land suitability and zonation of commodities in Rasau Jaya Dua Village.

The research location is in Rasau Jaya Dua Village, Kubu Raya Regency, West Kalimantan (Fig. 4) [13]. The evaluation of land suitability and commodity zoning in Rasau Jaya Dua Village is presented in Fig. 5 [13].

Source [13]:

Source [13]:

Rasau Jaya Dua Village has enormous agricultural business potential. It consists almost entirely of tidal alluvial deposits located in alluvium formations and swamp deposits which are mud and peat formations. Based on soil chemical and physical properties, soil type in the research location is classified as Histosols (Trophohemists) or peat soil (Fig. 4) [13], and the land use is suitable for maize, pineapple, and cassava (Fig. 5) [13]. This type of soil comes from organic parent materials such as from swamp forests or swamp grasses, more than 0.5 m soil thickness, brown to blackish color, loamy dust soil texture, not sticky to a bit sticky soil consistency, more than 30 % organic content, generally very acidic (pH 4.0), and low nutrient content. In accordance with land use and commodity zoning results, Rasau Jaya Dua Village is mostly dominated by maize, cassava, and a small part of rice crops (Fig. 5).

### 3.2 Existing condition in the study area

In Rasau Jaya Dua Village, there is a farmer groups association called 'Sri Rejeki' which consists of 31 farmer groups with an average of 18 members per farmer group, so there are approximately 558 farmers. There is one livestock farming group, namely “Kersa Usaha,” while the rest farmer groups cultivate maize, rice, and horticulture crops. The area of peatland planted with maize is about 300 ha with a rice-maize-vegetable or maize-vegetable-vegetable cropping pattern. The average maize productivity is 4 t ha^−1^ and the number of cattle is 48 heads. Cattle fodder is obtained from cutting grass in the forest and from fresh corn waste. Generally, farmers look for grass fodder twice a day (morning and afternoon). Cultivation of corn and cattle farming are not well integrated. Corn waste is used as fresh animal feed with low nutrients content, while both solid and liquid livestock waste (urine) is not properly managed. It needs to be appropriately utilized as organic fertilizer for corn and vegetable cultivation. The average monthly farmers' income from cultivating corn and vegetables, and cattle farming is IDR 4,760,000. This income is used for daily living costs of farmers' household, such as food, transportation, children's education, and health fees.

### 3.3 Initiation and strengthening of farmer institutions

In the village of Rasau Jaya Dua, there is a farmer groups association called 'Sri Rejeki' which consists of 31 farmer groups with an average of 18 members per farmer group. In this research, social engineering of farmers’ institutions was carried out by providing coaching and guidance to farmer groups, including administration management, financial bookkeeping, savings and loan activities, management of agricultural production facilities kiosks, and marketing management.

**Fig. 6:**
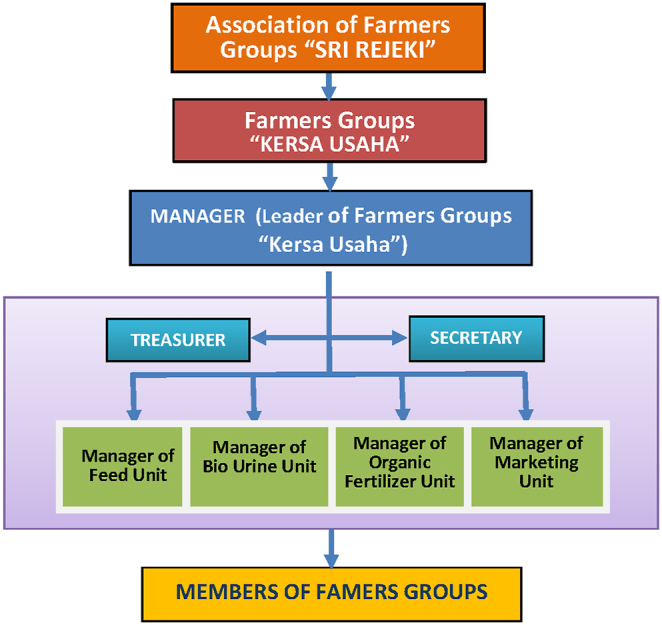
Organizational structure of working groups in Rasau Jaya Dua Village.

Institutional initiation was carried out by the establishment of a working group assisting the farmer groups association "Sri Rejeki" and collaborating with the farmer group "Kersa Usaha" in managing production and marketing the results of integration activities of maize and cattle (Fig. 6).

### 3.4 Increasing maize productivity on the peatlands

**Fig. 7:**
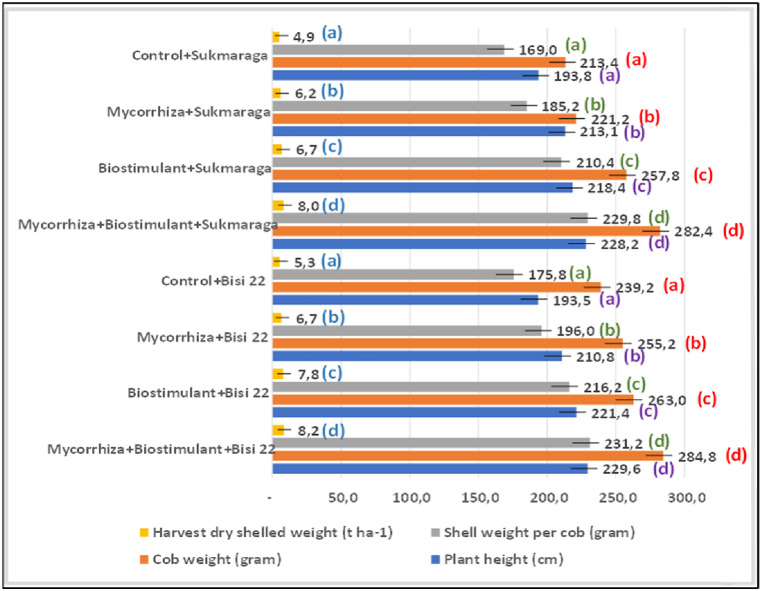
The effect of mycorrhiza and biostimulants on the growth and productivity of Sukmaraga and Bisi 22 maize varieties on the peatlands in Rasau Jaya Dua Village. Means of the numbers followed by the same letter is not significantly different on 5 % Duncan's Multiple Range test.

The results of research on maize cultivation on the peatlands with mycorrhizal biofertilizer and biostimulants from seaweed extract on Sukmaraga maize variety (inhybride) yielded 8.24 t ha^−1^ or an increase of 62.85 % from the control treatment, while Bisi 22 maize variety (hybrid) yielded 8.0 t ha^−1^ or an increase of 56.42 % over the control (Fig. 7).

The application of mycorrhizae can affect root growth by strengthening lateral structure of the roots, increasing endogenous auxin and other compounds that can increase nutrient uptake to the roots, and thus it can lead to increased plant growth [20]. Mycorrhizal functions can increase the absorption of soil nutrients (N, P, K, Zn, Mg, Cu, and Ca), facilitate the availability of soil phosphates, and absorb soil nutrients fixation which are not available to the plants [21]. In addition, the application of mycorrhiza into peat soil can help decompose nutrients absorbed in soil colloid, especially P fixation with Al and Fe oxides in acid soils [22]. Mycorrhiza can absorb phosphate in the soil with external hyphae, which are immediately converted into polyphosphate compounds and broken down into organic phosphate that can be absorbed by plants [[23], [24], [25]]. Mycorrhiza increased the cob weight per plant of sweet maize by 6.82 kg plot^−1^ and enhanced composite corn kernel by 6.55 t ha^−1^ [26].

The benefit of seaweed extracting biostimulants is to improve the development of strong roots in plants and to absorb more nutrients and water from soil [27]. Seaweed extracts affect plant growth and increase yields and plant tolerance to abiotic and biotic stresses [28]. The application of seaweed extract to maize leaves can significantly absorb more micro-nutrients than control [29]. In addition, seaweed extract influenced the increased yield of maize kernels by 15 % and the increased number of seeds per cob and the longer cob [30]. Sufficient nutrients during maize growth activate the metabolic process, hence the elongation and division of cell differentiation increase, thereby enhancing maize seeds. This makes higher yields of Sukmaraga and Bisi 22, i.e. 8.24 and 8.0 t ha^−1^, respectively.

### 3.5 Cattle feed processing from maize waste

Maize biomass is classified as low-quality fresh forage for animal feed because it has high crude fiber content, low digestibility, and low nutrient content (5.56 % protein, 33.58 % crude fiber, 1.25 % crude fat, 7.28 ash, and carbohydrates in extract material without nitrogen (BETN) of 52.32 % [31]. Maize cultivation research was conducted to produce an average maize biomass of 7.2 t ha^−1^. The results showed that 100 kg maize biomass mixed with silage ingredients such as 2 kg brown sugar, 4 kg rice bran, and 15 mL probiotic supplement, could produce high-quality animal feed (Table 1). Animal feed produced by this research has a long shelf life and is preferred by cattle, in addition, it is efficient (Table 2).

**Table 1:** Analysis of silage as cattle feed in Rasau Jaya Dua Village.

Parameter		*Value		
		Silage A	Silage B	Silage C
pH	–	4.0	4.2	4.5
Phosphorus	(ppm)	50.87	52.59	53.93
Potassium	(ppm)	44.75	47,22	50.93
Fat	(%)	6.82	5.18	8.04
Proteins	(%)	5.44	5.85	6.91
Ammonia	(%)	2.88	4.18	6.66

Notes.

*A = 4 kg brown sugar +6 kg rice bran + 100 kg maize biomass + 15 mL supplement.

B = 3 kg brown sugar +5 kg rice bran + 100 kg maize biomass + 15 mL supplement.

C = 2 kg brown sugar + 4 kg rice bran + 100 kg maize biomass + 15 mL supplement.

**Table 2:** Analysis of efficiency and R/C ratio.

Descriptions		*Treatments		
		A	B	C
Silage price (IDR)		11.658	9.858	8.058
Forage price (IDR)		15.000	15.000	15.000
Efficiency (IDR)		3.342	5.142	6.942
Output				
Revenue	Price per kg of silage (IDR)	Price per 90 kg of silage (IDR)		
	1500	135,000	135,000	135,000
Profit (IDR)		18,417	36,417	54,417
R/C ratio		1.16	1.37	1.68

Notes.

*A = 4 kg brown sugar + 6 kg rice bran + 100 kg maize biomass + 15 mL supplement.

B = 3 kg brown sugar + 5 kg rice bran + 100 kg maize biomass + 15 mL supplement.

C = 2 kg brown sugar + 4 kg rice bran + 100 kg maize biomass + 15 mL supplement.

The results showed that fermented corn biomass could be utilized as high-quality and efficient animal feed, and most importantly is favored by cattle. Cattle feed silage can increase the weight of cows. The results showed that silage for cattle feeds with the formulation of 2 kg brown sugar, 4 kg rice bran, and 15 mL probiotic supplements, could increase the body weight of cattle by 0.8 kg head^−1^ day^−1^ or equivalent to an income of Rp. 18,000 head^−1^ day^−1^. While the cattle population increased by 15 % per year from 48 to 70 heads during the study in Rasau Jaya Dua Village.

### 3.6 Organic fertilizer processing from cattle waste

Cattle waste can be processed into solid and liquid organic fertilizers. The analysis of nutrient content in solid organic fertilizers at the study sites is shown in Table 3.

**Table 3:** Nutrient analysis of granule organic fertilizers in Rasau Jaya Dua Village.

Parameters		Value	Criteria*
pH	–	7.34	Slightly alkaline
Organic carbon	(%)	29.73	Very high
Total nitrogen	(%)	2.97	Very high
C/N ratio	–	10.01	Medium
Phosphorus	(%)	0.74	Medium
Potassium	(%)	1.51	High
Calcium	(%)	4.31	High
Magnesium	(%)	1.43	Medium

Notes: *Soil Research Institute of Indonesia.

The best treatment of liquid cow waste (urine) processing into biourine fertilizer, is cow urine added with bio-activator + liquid herbs made from ginger, turmeric, curcuma, aromatic ginger, lemongrass, and garlic (5 % amount of all ingredients) + rumen enzymes (1 L enzyme per 50 L urine), rice washing water (100 cc per L urine), potassium permanganate (KMnO_4_) and Titanium dioxide (TiO_2_) (each 2 g per L water). After the cow urine is fermented, the color changes to dark brown and the pungent odor in the cow urine reduces and turns into an herbal odor.

Nutrient content analysis of liquid organic fertilizer (biourine) is shown in Table 4.

**Table 4:** Nutrient content of cattle's biourine in Rasau Jaya Dua Village.

Parameters		*Treatment A		Treatment B		Treatment C	
pH	–	5.25	Slightly acidic	5.75	Slightly acidic	7.75	Slightly alkaline
Organic carbon	(%)	1.01	Low	1.09	Low	1.29	Low
Total nitrogen	(%)	2.03	Medium	2.01	Medium	3.06	Medium
Ratio C/N	–	0.49	Very low	0.54	Very low	0,63	Very low
Phosphorus	(ppm)	5.24	Low	6.11	Low	12,14	Medium
Potassium	(ppm)	52.20	High	91.26	Very high	2385,24	Very high
Calcium	(ppm)	32.15	Very high	36.32	Very high	71,23	Very high
Magnesium	(ppm)	45.12	Very high	79,65	Very high	378,72	Very high
Smell	–	Smell	–	Smell	–	No smell	–
Color	–	Brown	–	Brown	–	Dark brown	–

Notes: *.

A) Cow urine + bio-activator.

B) Cow urine + bio-activator + liquid herbs (ginger, turmeric, curcuma, aromatic ginger, lemongrass, and garlic (5 % amount of all ingredients) + enzymes from the rumen (1 L enzyme per 50 L urine).

C) Cow urine + bio-activator + liquid herbs (ginger, turmeric, curcuma, aromatic ginger, lemongrass, and garlic (5 % amount of all ingredients) + enzymes from the rumen (1 L enzyme per 50 L urine) + rice washing water (100 mL rice washing water per L urine) + potassium permanganate (KMnO_4_) and Titanium dioxide (TiO_2_) (2 g each per L water).

### 3.7 Financial feasibility of maize cultivation on the peatlands

In accordance with the results of ameliorant studies on corn cultivated in the peatlands conducted in Rasau Jaya Dua Village, circular agriculture based on the integration of maize and cattle livestock is very relevant and can be recommended as an alternative to increase sustainable and environment-friendly farmer income. Based on financial analysis results, several ameliorants application on corn plants can provide benefits and is feasible with an R/C ratio value between 1.01 and 1.42 (Table 5) [32].

**Table 5:** Financial analysis per hectare of maize demonstration plots on peatlands in Rasau Jaya Dua Village.

Description	Treatments*				
	A	B	C	D	E
Production facilities cost (IDR)	6,425,000	6,091,667	4,165,000	3,425,000	3,840,667
Labor wages cost (IDR)	3,220,000	3,220,000	3,220,000	3,220,000	3,220,000
Other cost (IDR)	1,478,750	1,478,750	1,478,750	1,478,750	1,478,750
Expenses cost (IDR)	13,550,400	10,915,200	12,751,200	9,662,400	12,124,800
Income (IDR)	11,123,750	10,790,417	8,863,750	8,123,750	8,539,417
Profit (IDR)	2,426,650	124,783	3,887,450	1,538,650	3,585,383
R/C ratio	1.22	1.01	1.44	1.19	1.42

Notes: *.

A. Ameliorant: 1 t ha^−1^ pugam, 300 kg ha^−1^ Urea, 150 kg ha^−1^ KCl, 100 kg ha^−1^ kieserite.

B. Ameliorant: 5 t ha^−1^ chicken manure, 300 kg ha^−1^ Urea, 200 kg ha^−1^ SP-36, 150 kg ha^−1^ KCl, 100 kg ha^−1^ kieserite, 15 kg ha^−1^ CuSO_4_.

C. Ameliorant: 1 t ha^−1^ dolomite, 300 kg ha^−1^ Urea, 200 kg ha^−1^ SP-36, 150 kg ha^−1^ KCl, 100 kg ha^−1^ kieserite, 15 kg ha^−1^ CuSO_4_.

D. Control: without ameliorant, 300 kg ha^−1^ Urea, 200 kg ha^−1^ SP-36, 150 kg ha^−1^ KCl, 100 kg ha^−1^ kieserite, 15 kg ha^−1^ CuSO_4_.

E. Control (farmer method): 1.6 t ha^−1^ dolomite, 8 L ha^−1^ Trichoderma, 1.25 t ha^−1^ chicken manure, 50 kg ha^−1^ Urea, 250 kg ha^−1^ NPK Phonska, 50 kg ha^−1^ KCl.

**Fig. 8:**
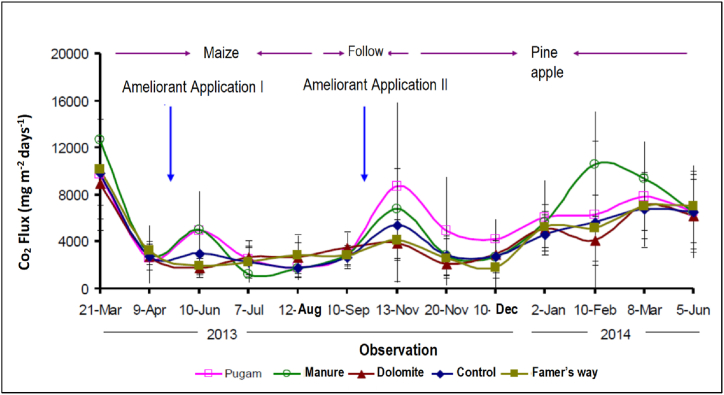
Pattern of CO_2_ flux in maize and pineapple plants on peatlands in Rasau Jaya Dua Village.

In addition to financial analysis, the results of greenhouse gas study on the peatlands in Rasau Jaya Dua Village showed that CO_2_ flux at the end of the rainy season was around 2600–12700 mg CO_2_ m^−2^ day^−1^ before the amelioration treatment, whereas, after the amelioration, it reduced CO_2_ flux during plant growth and increased again after the maize harvest (Fig. 8) [33].

CO_2_ flux tended to be higher during the growth of pineapple than corn (Fig. 8). Amelioration of "pugam" (peat fertilizer) was seen to produce high CO_2_ flux, especially during fallows and during the growth of pineapple plants. Management of peatlands by adding ameliorants reduced CO_2_ emissions by 34.92 %. CO_2_ emissions before the application of ameliorant as a baseline were higher than after the application of ameliorant and plants. In corn cultivation, the lowest CO_2_ emission was resulted from the farmer's treatment, i.e. 7.73 + 0.8 t CO_2_ ha^−1^ year^−1^, and the highest from the pugam treatment, i.e. 13.47 + 6.3 t CO_2_ ha^−1^ year^−1^. The lowest CO_2_ emissions in conditions without plants was from the control treatment (7.97 + 2.3 t CO_2_ ha^−1^ year^−1^) and the highest from the chicken manure treatment (11.14 + 2.1 t CO_2_ ha^−1^ year^−1^) (Table 6) [34].

**Table 6:** Total CO_2_ gas emissions on different vegetation in Rasau Jaya Dua Village.

Treatments	CO_2_ emission (t CO_2_ ha^−1^ yr^−1^)			
	Baseline	Maize	Fallow	Pineapple
Peat fertilizer (Pugam)	22.75 ± 18.2	13.47 ± 6.3	8.49 ± 3.0	22.10 ± 6.1
Manure	28.51 ± 25.1	11.37 ± 9.6	8.32 ± 3.0	21.50 ± 11.1
Dolomite	21.37 ± 16.1	8.07 ± 2.2	11.14 ± 2.1	15.48 ± 6.5
Control	22.98 ± 18.2	9.69 ± 1.8	7.97 ± 2.3	17.85 ± 5.5
Farmer's way	24.49 ± 17.9	7.73 ± 0.8	10.33 ± 0.0	16.15 ± 7.4

Furthermore, it was reported that CO_2_ flux was higher in the ameliorant treatment to corn plants than without ameliorant (control). The highest CO_2_ emissions in corn plants occurred in the application of pugam” ameliorant. CO_2_ emissions on plots with chicken manure and peat fertilizer plots were higher than the control, while dolomite ameliorant or farmers' practices emitted CO_2_ lower than the control (Table 7) [33].

**Table 7:** CO_2_ flux of peatlands treated with ameliorant in Rasau Jaya Dua Village.

Ameliorant treatment	CO_2_ emission (t CO_2_ ha^−1^ season^−1^)	
	Maize^a^	Pineapple^a^
Control	2.2 ± 0.6	11.9 ± 5.0
Manure	2.4 ± 0.8	15.2 ± 4.4
Peat fertilizer (Pugam)	2.7 ± 1.0	15.3 ± 6.4
Dolomite	2.4 ± 0.5	10.8 ± 1.9
Farmer's way	2.2 ± 0.7	11.3 ± 1.9

a Maize harvest age 3 months and pineapple harvest age 8 months.

In relation to maize-cattle integration study, NO_2_ is released into the atmosphere throughout the composting process [35]. The formation of N can also affect NH_3_ emission from the livestock housing. Total CO_2_ emission is decreased by 55.86 % in the composting and vermi composting process due to C emission into the atmosphere in the form of CO_2_ and CH_4_ [35]. Overall, greenhouse gas (GHGs) emission is reduced by 10 % if utilizing cow manure for composting and vermicomposting compared with using the manure for aerobic digestion (biogas) [35].

### 3.8 Recommendations for the development of rural circular agriculture in the integration system of maize and cattle farming on peatlands

**Fig. 9:**
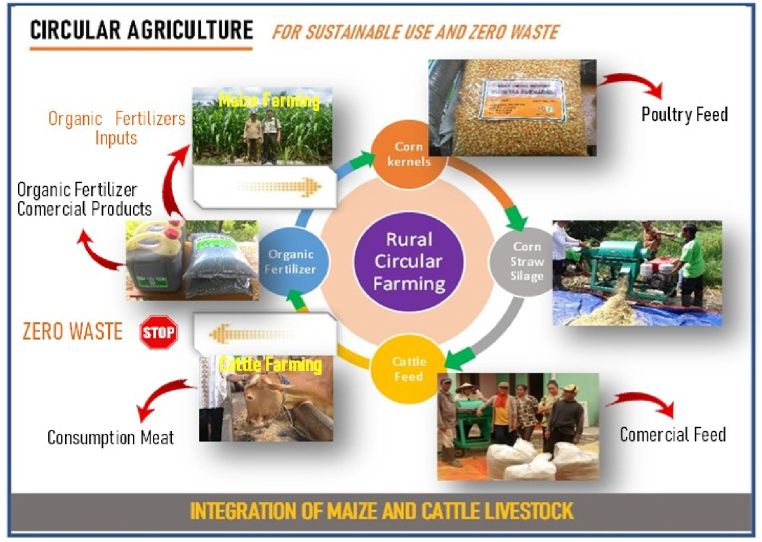
Circular agriculture of integration of maize and cattle livestock in Rasau Jaya Dua Village.

The integration system between maize and cattle in this study reduces the risk of mono-culture farming failure. This integration system is implemented with the principle of circular agriculture, namely an agricultural system that can utilize local resources by utilizing waste (zero waste) produced from both corn and cattle. The waste is processed to make products that can be used as input for production facilities for corn and cattle, such as for cattle feed and organic fertilizer (Fig. 9).

The development of a rural circular agriculture model based on the integration of maize and cattle in peatlands in Rasau Jaya Dua Village, Kubu Raya Regency, West Kalimantan is not a partial model that only involves the biophysical factors of the land, but it is a comprehensive model involving many factors such as socio-economic farmers, farmer institutions and stakeholders. This affects the successful maize and cattle integration system that is sustainable and environmentally friendly. In this circular agriculture system, high-quality cattle feed and organic fertilizer become added values. This circular agriculture system can also reduce environmental pollution from corn and cattle waste. This model involves farmer groups and farmer groups association, researchers and extension workers, government agencies, the private sector, and banking through technological innovation and institutional engineering.

**Fig. 10:**
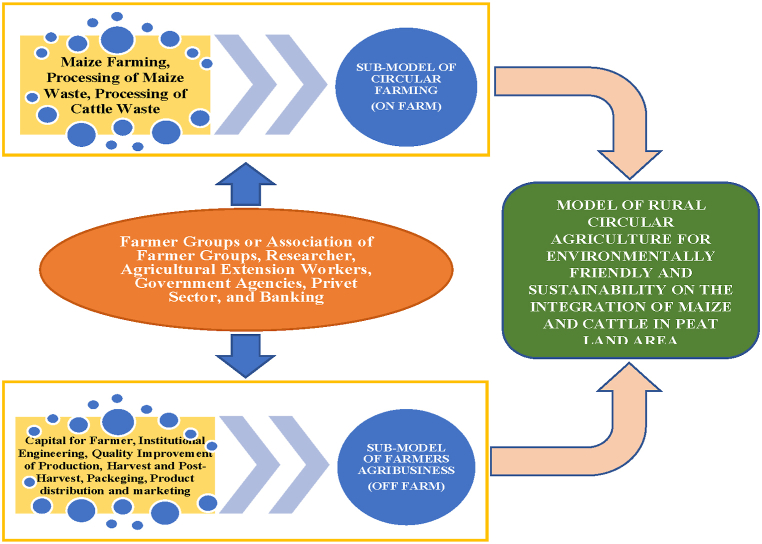
Circular agriculture model on maize and cattle integration in peatlands in West Kalimantan, Indonesia.

This model is packaged in an on-farm rural agro-industrial sub-model and an off-farm rural agribusiness institutional sub-model, starting from corn cultivation, processing corn waste into cattle feed, processing cattle waste into solid and liquid organic fertilizer, capital generated from farmers’ institutional engineering, pre-production, production, harvest and post-harvest subsystems as well as distribution and marketing of products supported by banks. The recommended model for development of a rural bioindustry system for corn and cattle integration on the peatlands in West Kalimantan is shown in Fig. 10.

Different types of farming combination offer some advantages, for instance minimizing environmental pollution and generating income, and using the recommended processes in particular cow manure, crop solid waste, and related substances would enhance profits for the family farm [35]. Utilization of vermi compost, cow-washing wastewater, coupling cow breeding with crop cultivation in an integrated system improve soil fertility, enhance crop growth, reduce water and soil contamination and the amount of greenhouse gas emissions, conserve the environment, and in particular, increasing the family farms’ income [35].

In the integrated agricultural system, composting is made from cow dung and crop solid waste. It can lower greenhouse gas emissions while also producing organic fertilizer to provide nutrients for plant growth. In this way, the primary greenhouse gas components C and N found in cow dung are transformed into organic material forms. Because they transform into stable forms, N and C are not easily released into the atmosphere [36]. Then, these provide plants with nutrients, enhancing the soil's quality and boosting the plantation soil's ability to store water. Furthermore, less chemical fertilizers, especially N fertilizers, will drastically lower the amount of greenhouse gases generated. Through soil carbon (C) sequestration, organic C addition to the soil may be able to balance greenhouse gas emissions [37]. As a result, a net C sink would result from the last step [37].

Nutrient conversion occurs throughout the composting process of cow manure, including N, P, K, and C. P and K contents rise since organic components mineralize throughout the composting process [38,39]. The quantity of P and K that are released in composting and vermicomposting products for plants to absorb is enhanced by the higher amounts of P and K obtained from these processes, which are also vital nutrients for food production [35,38].

The heat produced by the composting process also minimizes odor. In addition, germs including Salmonella, Coliforms, and *E. coli* are eradicated [38,39]. When compost is applied on the soil, the increased population of beneficial bacteria that results from composting offers additional advantages. As composting proceeds, the overall N concentration decreases from 1.75 % to 1.07 % [38,39].

Adoption of integrated crop-livestock system (ICLS) reduces agricultural activity risk. Risk analysis research concluded that it was advantageous for ICLS products to be diversified [40,41]. Nevertheless, the system was more complicated because it called for the producer to possess a wider range of technical and commercial understanding of livestock- and agriculture-related activities. Furthermore, it was thought that ICLS was less susceptible to changes in market and operational variables [40,41].

Compared to the current conventional system, the ICLS is more economically feasible [40,41]. In comparison to agricultural systems, the risks associated with the integrated systems were lower [40,41]. But of all the systems examined, the cattle system had the lowest risk. The ICLS requires less area than the conventional system to achieve the break-even threshold [40,41].

It is acknowledged that alternative combinations of integrated systems can be investigated, and that working with grain rotations in the same region such as soybean-corn can be done to avoid limiting corn output to just one type of crop.

## 4 Conclusion and policy implication

### 4.1 Conclusion

The development of a rural circular agriculture model based on maize and cattle integration system is very prospective in meeting the demand for cattle feed and organic fertilizer. This farming integration could be the forerunner to agribusiness development based on food crops and ruminants, especially in West Kalimantan Province. Based on the potential and carrying capacity, maize waste can provide feed for cattle, and cattle waste can be used as input in maize cultivation as organic fertilizer.

Derivative products that can be developed from the circular agriculture process in the maize cattle integration system are chicken feed (shelled corn), ruminant animal feed (silage), and organic fertilizers. Cattle waste is used to make solid and liquid organic fertilizer which can increase maize and horticultural crops productivity in addition to be commercially viable in the market, thereby increasing farmers' income.

Institutional social engineering such as a rural agribusiness institutional working group model facilitates the activities of circular agricultural development in maize and cattle integration. Rural circular agriculture based on maize and cattle integration is still less practised, and hence this model is recommended to be implemented and scaled up on other maize farming and cattle farms in the surrounding peatland area in West Kalimantan. Future research of this model might be carried out in the dryland area in West Kalimantan that has potential for maize and cattle farming. We also advise employing optimization tools, like financial models for the future research, since they enable the investigation of ways to generate financial benefits while considering the ideal size for cultivated areas, technologies for the available resources, and production goals.

### 4.2 Policy implication

Advice for central and local governments that have large peatlands to make policies for developing and managing peatlands with green economy programs in rural areas. The policy can be implemented in accordance with the model obtained from this research, namely technological innovations in circular agriculture model based on maize and cattle integration system. Taking that into consideration, it is expected that family farm income will increase, and the agricultural innovation technology is environment-friendly and sustainable; and eventually, it can enhance the economy in rural areas.

The development of circular agriculture on maize and cattle integration based can be established more intensively by developing products derived from maize and cattle through food technology and home industries, such as corn rice, flour, starch, corn oil, bioethanol, canned beef, and shredded beef. It is necessary to encourage coordination and collaboration with stakeholders at the provincial and district levels and entrepreneurs in order to develop and strengthen the rural circular agriculture model that can support national and regional food security.

## Data availability

The data that support the findings of this study are available upon reasonable request.

## Ethics approval

The study holds no ethical community is involved.

## CRediT authorship contribution statement

**Dwi P. Widiastuti:** Writing – review & editing, Writing – original draft, Visualization, Validation, Project administration, Methodology, Investigation, Funding acquisition, Formal analysis, Data curation, Conceptualization. **Muhammad Hatta:** Writing – review & editing, Writing – original draft, Visualization, Validation, Project administration, Methodology, Investigation, Funding acquisition, Formal analysis, Data curation, Conceptualization. **Hozin Aziz:** Visualization, Validation, Software, Resources, Investigation. **Dadan Permana:** Visualization, Validation, Supervision, Software, Resources. **Putri Tria Santari:** Visualization, Validation, Supervision, Software, Project administration. **Eni Siti Rohaeni:** Writing – review & editing, Writing – original draft, Visualization, Validation, Supervision, Methodology, Investigation, Formal analysis, Data curation, Conceptualization. **Salfina Nurdin Ahmad:** Writing – review & editing, Writing – original draft, Visualization, Validation, Supervision, Methodology, Investigation, Formal analysis, Data curation, Conceptualization. **Bachtar Bakrie:** Writing – review & editing, Writing – original draft, Visualization, Validation, Supervision, Methodology, Investigation, Formal analysis, Data curation, Conceptualization. **Siti Sehat Tan:** . **Susana I.W. Rakhmani:** Writing – review & editing, Writing – original draft, Visualization, Validation, Supervision, Methodology, Investigation, Formal analysis, Data curation, Conceptualization.

## Declaration of competing interest

The authors declare that they have no known competing financial interests or personal relationships that could have appeared to influence the work reported in this paper.

## References

[1] Ministry of Agrarian Affairs and Spatial Planning/National Land Agency. Stipulation of the Minister of Agrarian and Spatial Planning/Head of the National Land Agency of the Republic of Indonesia Number: 339/2018 Dated 8 October 2018 Concerning the Area of Standard Rice Fields in Indonesia.

[2] Indonesian Center for Agricultural Land Resources Research (2020).

[3] Sabiham S. (2010). Proc. Of Int. Workshop on Evaluation and Sustainable Management of Soil Carbon Sequestration in Asian Countries.

[4] Sabiham S., Tarigan S.D., Hariyadi Irsal Las, Agus F., Sukarman P., Setyanto Wahyunto (2012). Organic carbon storage and management strategies for reducing carbon emission from peatlands: a case study in oil palm plantations in West and Central Kalimantan, Indonesia. Pedologist.

[5] Masud M.M., Moniruzzaman M., Rashid M.M. (2011). Management and conservation of organic peat soils for sustainable crop production in Bangladesh. Bull. Inst. Trop. Agric. Kyushu Univ..

[6] Abat M., McLaughlin M.J., Kirby J.K., Stacey S.P. (2012). Adsorption and desorption of copper and zinc intropical peat soils of Sarawak Malaysia. Geoderma.

[7] Ratmini N.P.S., Herwenita, Irsan F. (2021). Climate change mitigation through superior varieties use to increase rice production in tidal swamp land. 6th International Conference on Climate Change 2021. IOP Conf. Ser. Earth Environ. Sci..

[8] Noor M., Nursyamsi D., Alwi M., Fahmi A. (2014). Prospects for sustainable agriculture in peatland: from farmer to researcher and researcher to farmer. Journal of Land Resources.

[9] Ratnayake A.S. (2020). Characteristics of lowland tropical peatlands: formation, classification, and decomposition. J. Trop. For. Environ..

[10] Food and Agriculture Organization (2023).

[11] Masruroh N., Fardian I., Febriyanti N., Muflihin M.D., Supriyanti S.S., Islami P.Y.N., Ilmiah D., Anas A.T., Panggiarti E.K., Honggowati S., Arifah S., Aziz A., Mualimin J., Wusqo U., Sujono R.I., Layli M., Amrina D.H., Firdaus B.M.A., RitongaNurhayati I., Widyawati R.F., Sari D.P., Widayanti I., Susetyo A.B., Sari S.W.H.P., Martutiningrum D., Romli N.A., Nurpratiwi S., FauziMahmudin M., Dahlan R. (2022).

[12] Statistics Indonesia, Statistical Yearbook of Indonesia 2020 (2020).

[13] Hatta M., KrisnohadiHartono A., Permana D. (2015).

[14] Gomez, Kwanchai A., Gomez A.A. (1984). An International Rice Research Institute Book.

[15] Briceño-Domínguez D., Hernández-Carmona G., Moyo M., Stirk W., van Staden J. (2014). Plant growth promoting activity of seaweed liquid extracts produced from Macrocystis pyrifera under different pH and temperature conditions. J. Appl. Phycol..

[16] Sulakhudin, Hatta M., Suryadi U.E. (2019). Application of coastal sediment and foliar seaweed extract and its influence to soil properties, growth and yield of shallot in peatland. Agrivita.

[17] Soekartawi (2002).

[18] Adnyana M.O., Kariyasa K. (1995). Competitive advantage model as an analytical tool in selecting superior agricultural commodities. Informatika Penelitian.

[19] Kay Ronald D., Edwards William M. (1994).

[20] Al-Juthery H.W.A., Drebee H.A., Al-Khafaji B.M.K., Hadi R.F. (2020). Plant biostimulants, seaweeds extract as a model (article review). IOP Conf. Ser. Earth Environ. Sci..

[21] Etesami H., Jeong B.R., Glick B.R. (2021). Contribution of arbuscular mycorrhizal fungi, phosphate-solubilizing bacteria, and silicon to p uptake by plant. Front. Plant Sci..

[22] Nuraini L., Lukiwati D.R., Fuskhah E. (2022). Growth response and yield of soybean (*Glycine max* (L.) Merr.) due to arbuscular mycorrhizal fungus (ACM) inoculation and natural phosphate fertilization. Agroplasma Journal.

[23] Farida R., Chozin M.A. (2015). The effect of arbuscular mycorrhizal fungi (CMA) and chicken manure doses on the growth and production of maize (*Zea mays* L.). Agrohorti Bulletin.

[24] Marlina N., Amir N. (2019). Proceedings of Environmentally Friendly Smart Farming for Farmer Welfare.

[25] UtamiHerlinawati C.D., Rosdiana E. (2021). Applications of bio-fertilizers microrrhiza and some types of green fertilizer on the yields of soybean (*Glycine max* L.). Agri.

[26] Abidin M., Darwanto S., Andayani R.D. (2017). Effect of doses of petroganic and mycorrhizal organic fertilizers on the growth and production of the talent variety of sweet corn (*Zea mays* saccharata). Hijau Cendikia Journal.

[27] Altindal D. (2019). Effects of seaweed extraction (SE) applications on seed germination characteristics of wheat in salinity conditions. International Journal Agriculture Forestry Life Sciences.

[28] Paradikovi N., Tekli T., Zeljkovi S., Lisjak M., Špoljarević M. (2019). Biostimulants research in some horticultural plant species. Food Energy Secur..

[29] Ertani A., Francioso O., Tinti A., Schiavon M., Pizzeghello D., Nardi S. (2018). Evaluation of seaweed extracts from laminaria and Ascophyllum nodosum Spp. as biostimulants in *Zea mays* L. using a combination of chemical, biochemical and morphological approaches. Front. Plant Sci..

[30] Trivedi K., Vijay A.K.G., Vaghela P., Ghosh A. (2018). Differential growth, yield, and biochemical responses of maize to the exogenous application of Kappaphycus alvarezii seaweed extract, at the grain filling stage under normal and drought conditions. Algal Res..

[31] Longland A.C. (2012). National assessment of forage quality. Forages and grazing in house nutrition.

[32] SugiartiJafri T., Kilmanun J.C. (18–19 August 2014). Proceedings of the National Seminar on Sustainable Management of Degraded Peatlands for Mitigating Greenhouse Gas Emissions and Increasing Economic Value.

[33] Setyanto P., Sopiawati T., Adriani T.A., Pramono A., Hervani A., Wahyuni S., Wihardjaka A. (18–19 August 2014). Proceedings of the National Seminar on Sustainable Management of Degraded Peatlands for Mitigating Greenhouse Gas Emissions and Increasing Economic Value.

[34] Sopiawati T., Wihardjaka A., Setyanto P., Sugiarti T. (18–19 August 2014). Proceedings of the National Seminar on Sustainable Management of Degraded Peatlands for Mitigating Greenhouse Gas Emissions and Increasing Economic Value.

[35] Hai L.T., Tran Q.B., Tra V.T., Nguyen T.P.T., Le T.N., Schnitzer H., Braunegg G., Le S., Hoang C.T., Nguyen X.C., Nguyen V.-H., Peng W., Kim S.Y., Lam S.S., Le Q.V. (2020). Integrated farming system producing zero emissions and sustainable livelihood for small-scale cattle farms: case study in the Mekong Delta, Vietnam. Environ. Pollut..

[36] Shuokr Q.A., Imad A.O., Jwan S.M. (2018). Design and study for composting process site. International Journal of Engineering Inventions.

[37] Stanley P.L., Rowntree J.E., Beede D.K., DeLonge M.S., Hamm M.W. (2018). Impacts of soil carbon sequestration on life cycle greenhouse gas emissions in Mid Western USA beef finishing systems. Agric. Syst..

[38] Suthar S., Pandey B., Gusain R., Gaur R.Z., Kumar K. (2017). Nutrient changes and biodynamics of *Eisenia fetida* during vermicomposting of water lettuce (*Pistia* sp.) biomass: a noxious weed of aquatic system. Environ. Sci. Pollut. Control Ser..

[39] Negi R., Suthar S. (2018). Degradation of paper mill wastewater sludge and cow dung by brown-rot fungi Oligoporus placenta and earthworm (*Eisenia fetida*) during vermicomposting. Cleaner Production.

[40] Ryschawy J., Choisis N., Choisis J.P., Joannon A., Gibon A. (2012). Mixed crop-livestock systems: an economic and environmental-friendly way of farming?. Animal.

[41] Simili F.F., Mendonça G.G., Gameiro A.H., Augusto J.G., Oliveira J.G., Menegatto L.S., Santos D.F.L. (2023). The economic value of sustainability of the integrated crop-livestock system in relation to conventional systems. Rev. Bras. Zootec..

